# Supplementary material for: Quantitative prediction of conditional vulnerabilities in regulatory and metabolic networks using PRIME
Source: NPJ Syst Biol Appl. 2021 Dec 6;7:43. doi: 10.1038/s41540-021-00205-6 (PMC8648758; doi:10.1038/s41540-021-00205-6)
Supplement: Supplementary file 1 — Supplementary Information [file 41540_2021_205_MOESM1_ESM.pdf]

Supporting information for the manuscript:

Quantitative prediction of conditional vulnerabilities in regulatory and metabolic networks using PRIME

Selva Rupa Christinal Immanuel[1], Mario L. Arrieta-Ortiz[1], Rene A. Ruiz[1], Min Pan[1], Adrian Lopez Garcia de Lomana[1], Eliza J.R. Peterson[1*], Nitin S. Baliga[1,2,3,4,*]

[1] Institute for Systems Biology, Seattle, WA, USA
[2] Departments of Biology and Microbiology, University of Washington, Seattle, WA, USA
[3] Molecular and Cellular Biology Program, University of Washington, Seattle, WA, USA
[4] Lawrence Berkeley National Lab, Berkeley, CA, USA
* Corresponding author
Eliza J.R. Peterson: eliza.peterson@isbscience.org
Nitin S. Baliga: nitin.baliga@isbscience.org (lead contact)

List of supplementary files:

1. **Supplementary figures:** Supplementary Figure 1 to Figure 10 **(this .pdf file)**
2. **Supplementary Table 1:** Summary of all variables used in the PRIME equations. **(at the end of this .pdf file)**
3. **Supplementary Data 1:** Gene expression compendium used for developing EGRIN models. (.xlsx file)
4. **Supplementary Data 2:** EGRIN and EGRIN-PD regulatory networks. (.xlsx file)
5. **Supplementary Data 3:** Details of combinatorial regulation in regulatory network. (.xlsx file)
6. **Supplementary Data 4:** Essential and non-essential predictions from PROM, IDREAM and PRIME for glycerol and cholesterol. (.xlsx file)
7. **Supplementary Data 5:** Drug treated transcriptomes after processing through DESeq2. (.xlsx file)
8. **Supplementary Data 6:** Essential and non-essential predictions from PRIME for isoniazid predictions. (.xlsx file)
9. **Supplementary Data 7:** Input files for PRIME used in this study (glycerol, cholesterol and INH models; gene expression compendium; beta values) - .mat file to use with MATLAB.

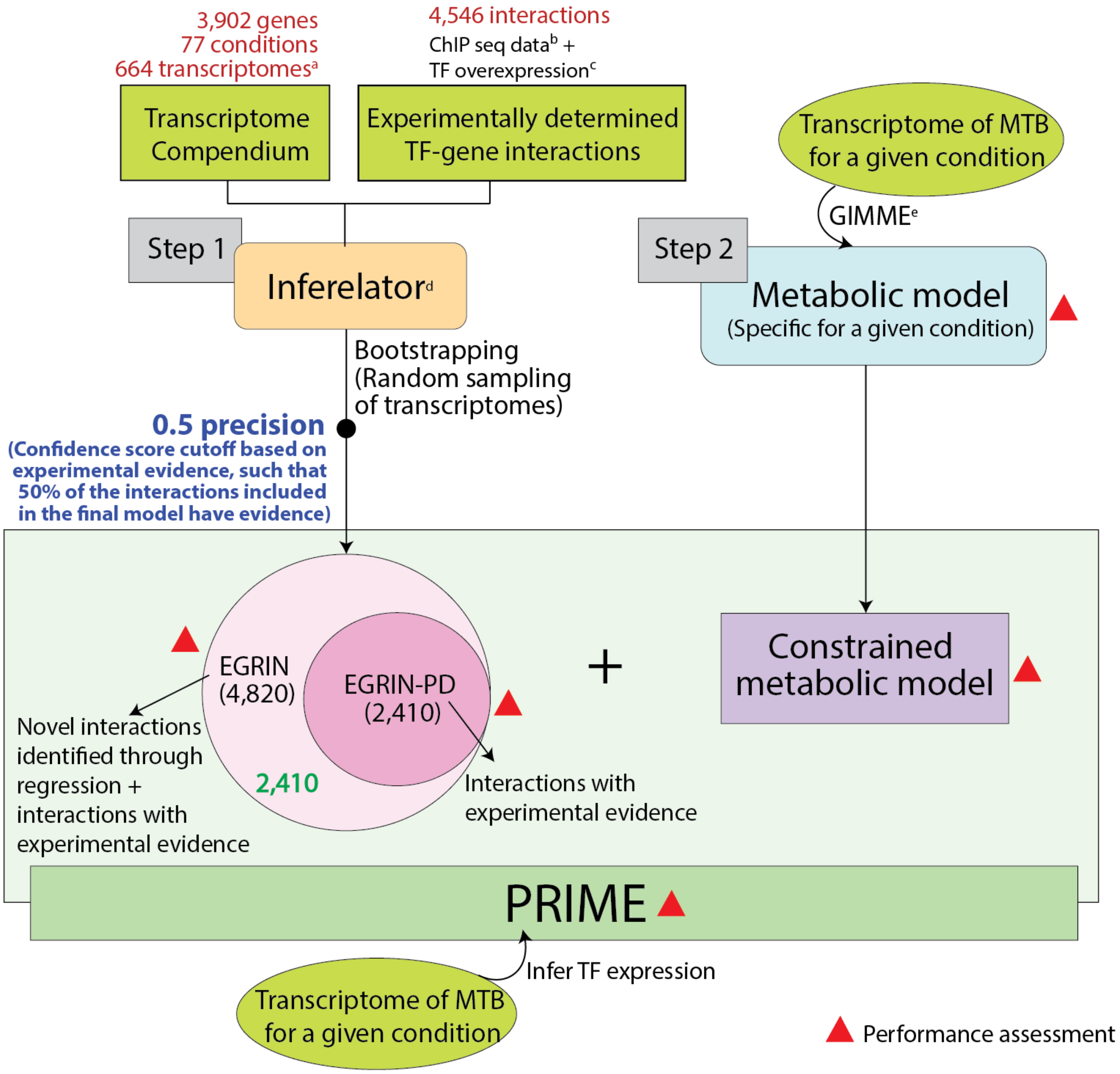

Supplementary Figure 1. **Schematic of data requirements and methodology for developing and using PRIME.** In step 1, the Inferelator algorithm uses a transcriptome compendium and experimentally determined TF-gene interactions as inputs to infer the EGRIN network. In step 2, the GIMME algorithm uses transcriptome profile from a given condition to constrain the metabolic network. PRIME then estimates condition-specific weights for the influence of TF(s) on regulation of downstream target metabolic gene(s) and the reaction(s) they catalyze to integrate EGRIN with the constrained metabolic network and predict environment-specific phenotypes. [a]-COLOMBOS database (http://colombos.net/) Moretto et al 2016; [b]- Minch et al 2015; [c]- Rustad et al 2014; [d]- Bonneau et al 2006; [e]- Becker et al 2008.

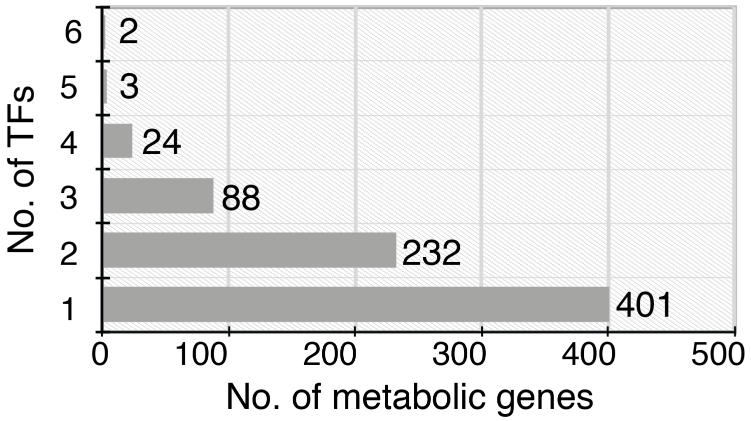

Supplementary Figure 2. Breakdown of numbers of metabolic genes based on the number of TFs implicated in their regulation.

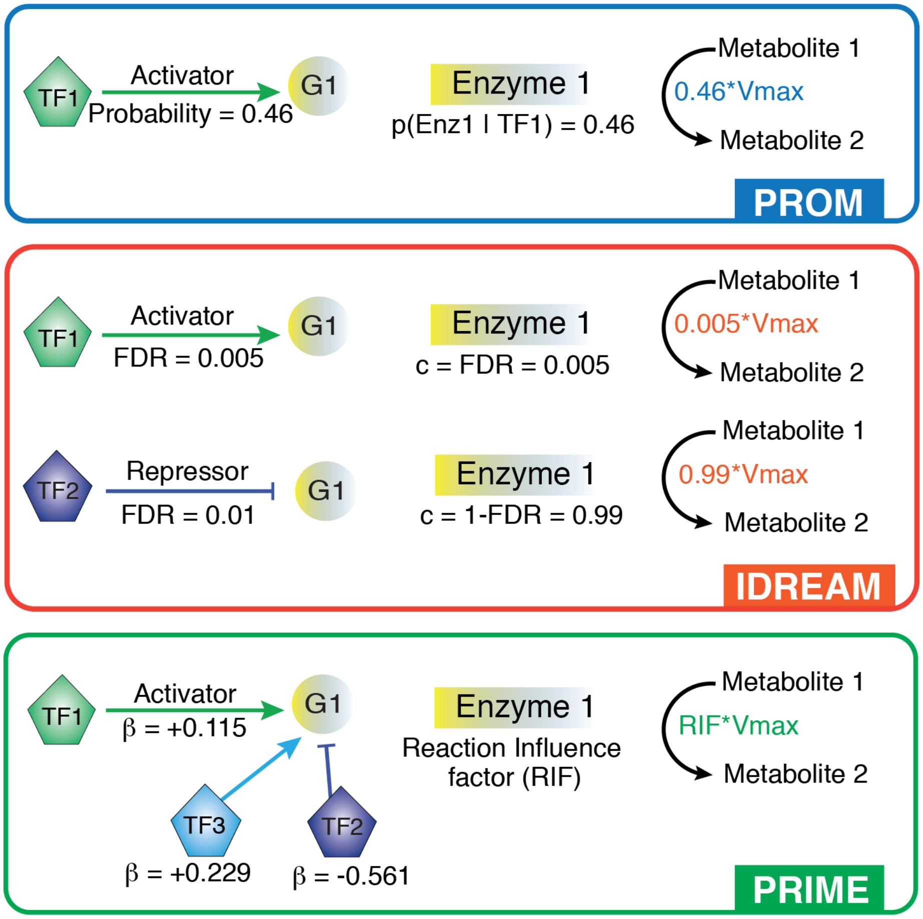

Supplementary Figure 3. Comparison of methodology used to integrate regulation and metabolism in PROM, IDREAM and PRIME. In PROM, the on/off state of the target metabolic gene of a TF is determined based on probabilities (p) pf TF-gene interactions estimated using a P-D interaction map and gene expression data. These probabilities are then applied on the reaction flux. IDREAM uses FDRs from EGRIN in place of probabilities. If a TF is an activator, the FDR values are directly applied on the reaction flux. If the TF is a repressor, (1-FDR) value is applied on the reaction flux. PRIME uses a reaction influence factor (RIF) that accounts for (i) weights (β) of the TF-gene interactions, (ii) TF expression and (iii) combinatorial regulation. We illustrate differences in methodologies used in PROM, IDREAM and PRIME with an example - ACALDi, a reaction catalyzed by the acetaldehyde dehydrogenase enzyme, which is encoded by Rv3535c. EGRIN predicted that Rv3535c is regulated by three TFs - Rv0047c, Rv1816 and Rv3574, with different weighted influences of 0.115, 0.229 and -0.561, respectively. The sign on these weights indicates that Rv0047 and Rv1816 are activators, whereas Rv3574 is a repressor. The RIF in PRIME accounts for combinatorial regulation of Rv3535c by all three TFs and their normalized absolute abundance to calculate relative quantitative influence of each TF. In this example, to predict the phenotypic consequence of knocking out Rv0047 on Mtb growth in the presence of glycerol, PRIME estimated RIF (0.9315) by dividing the weighted influence of Rv0047c (0.115) by the sum of influence weights of all three TFs (0.115 + 0.229 -0.561), multiplied to the scaled expression value of Rv0047. By multiplying this RIF to the upper bound of the flux

through ACALDi, PRIME updated flux bounds on the reaction catalyzed by Rv3535c. Based on this calculation PRIME predicted that knocking out Rv0047c would result in 7% decrease in flux through ACALDi, making this gene not essential for Mtb growth on glycerol. By contrast, based on the probability of Rv3535c regulation by Rv0047c (0.464), PROM predicted that knocking out Rv0047c would reduce ACALDi reaction flux by 56%, making it an essential gene. In this example, PRIME prediction was consistent with TnSeq findings, whereas PROM prediction was incorrect because it did not account for combinatorial regulation of Rv3535c by two additional TFs.

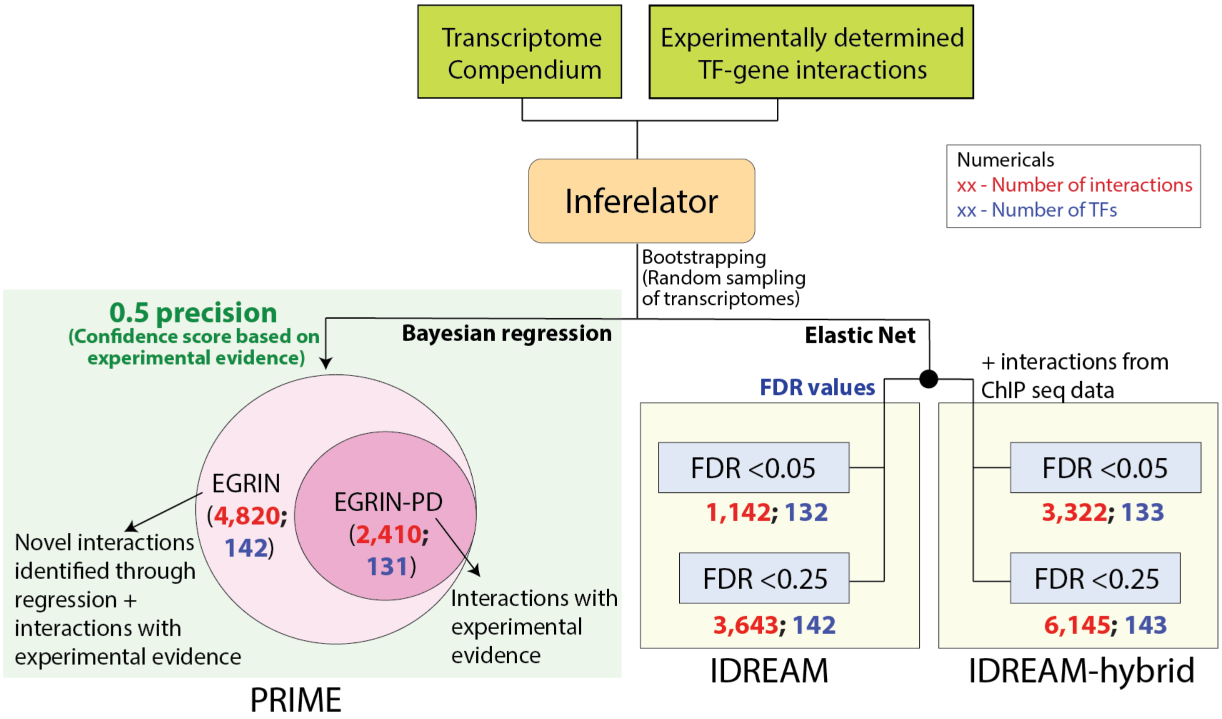

Supplementary Figure 4. **Flow chart depicting how regulatory networks were generated for IDREAM and PRIME.** IDREAM uses FDR values, calculated from multiple Inferelator runs on different subsets of the transcriptome compendium using a modified elastic net approach, to select TF-gene interactions. By contrast, PRIME uses TF-gene interactions from an EGRIN network inferred at 0.5 precision using a Bayesian regression, meaning half of all interactions recapitulate an experimentally derived network that was used as a prior in the inference procedure. The numbers indicated in red and blue fonts correspond to the number of TF-gene interactions and number of TFs in the networks, respectively. See **Supplementary Figure 1** for details about the transcriptome compendium and experimentally determined TF-gene interactions.

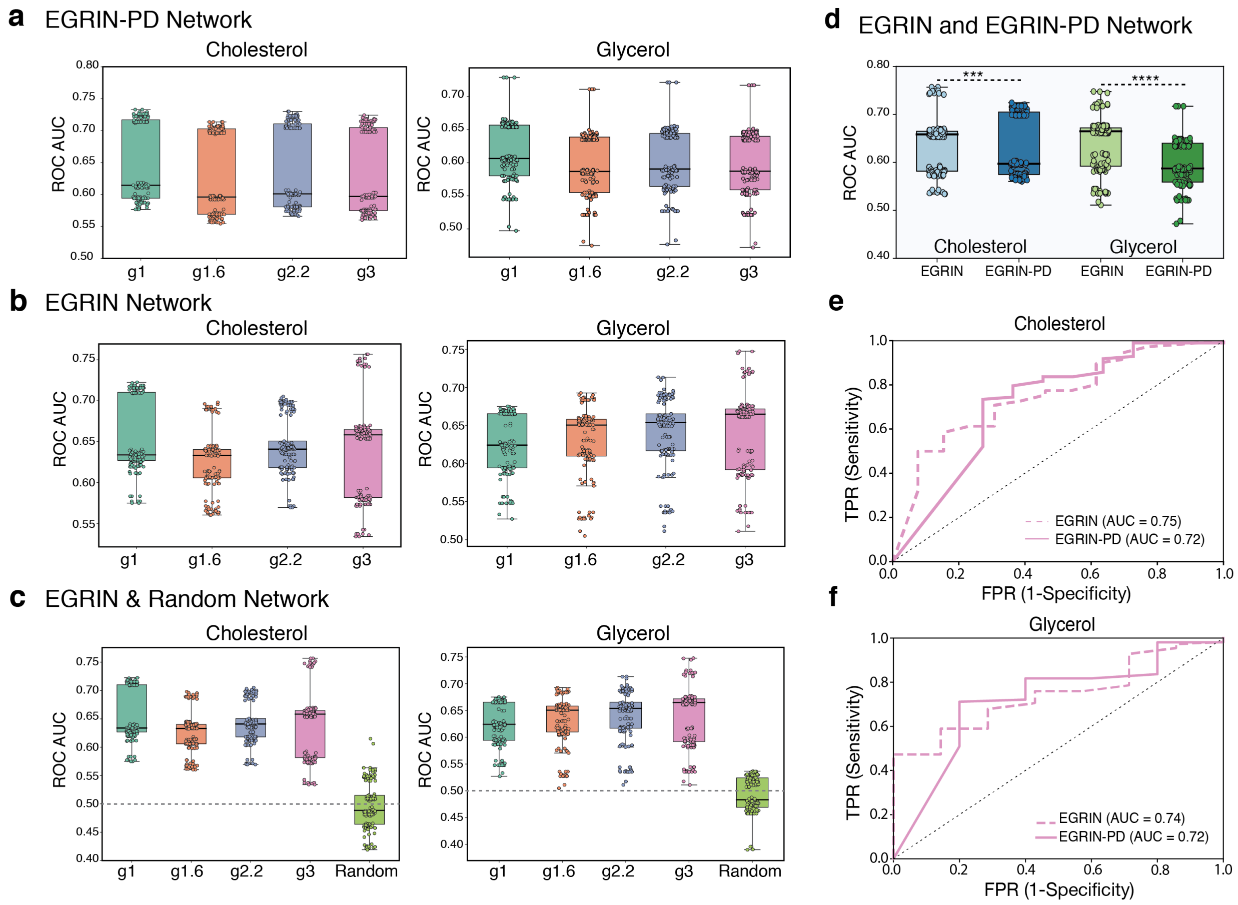

Supplementary Figure 5. Multiple EGRIN models were constructed, based on different values assigned to the "*g*" function in the Inferelator algorithm that defines how much of the prior weights has to be assigned while retrieving the network (see Bonneau et al 2006, Greenfield et al 2013, and Arrieta et al 2015 for more details and Methods). We generated networks for four values of *g* (1.1, 1.6, 2.2, and 3). The network provided in **Supplementary Table 2** has a *g* value of 3 and is the network used throughout the study. **a.** Network performance for EGRIN-PD network with all *g* values. **b.** Network performance for EGRIN network with all *g* values. **c.** Comparison of EGRIN performance with all *g* values and a random network. **d.** Comparison of performance between EGRIN and EGRIN-PD network in both (cholesterol and glycerol) growth conditions. Statistical significance was calculated as *p*-value with two sample t-test. *** p-value < 0.001 and **** p-value < 0.0001. All the boxes in the boxplot indicate the upper and lower quartiles of the data and the middle line is the median with the whiskers extending to 1.5× interquartile range. ROC curves for EGRIN and EGRIN P-D comparison in **e**. cholesterol and **f**. glycerol growth conditions.

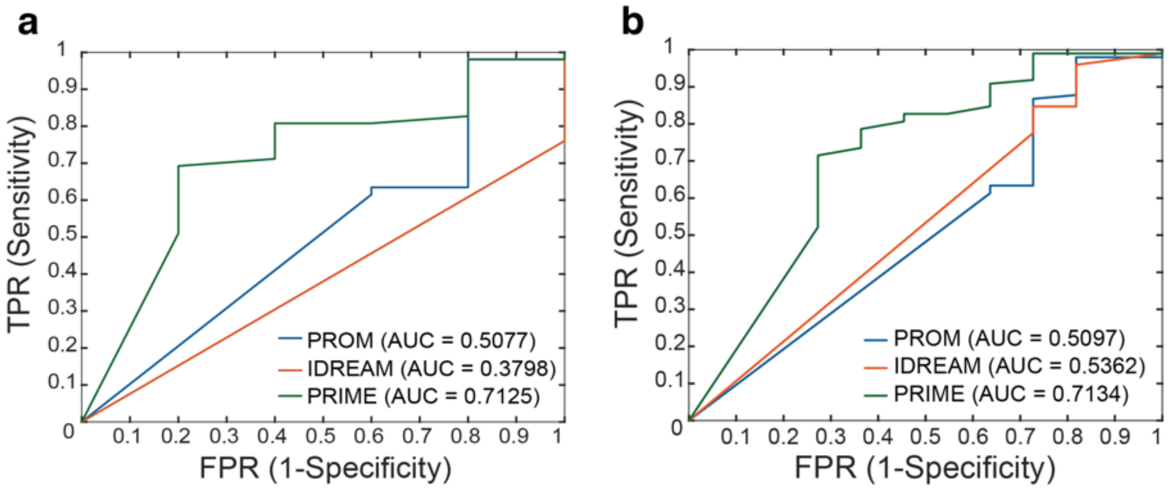

Supplementary Figure 6: ROC curve analysis for cholesterol and glycerol. Sensitivity and specificity curve for model predictions in **a.** cholesterol and **b**. glycerol.

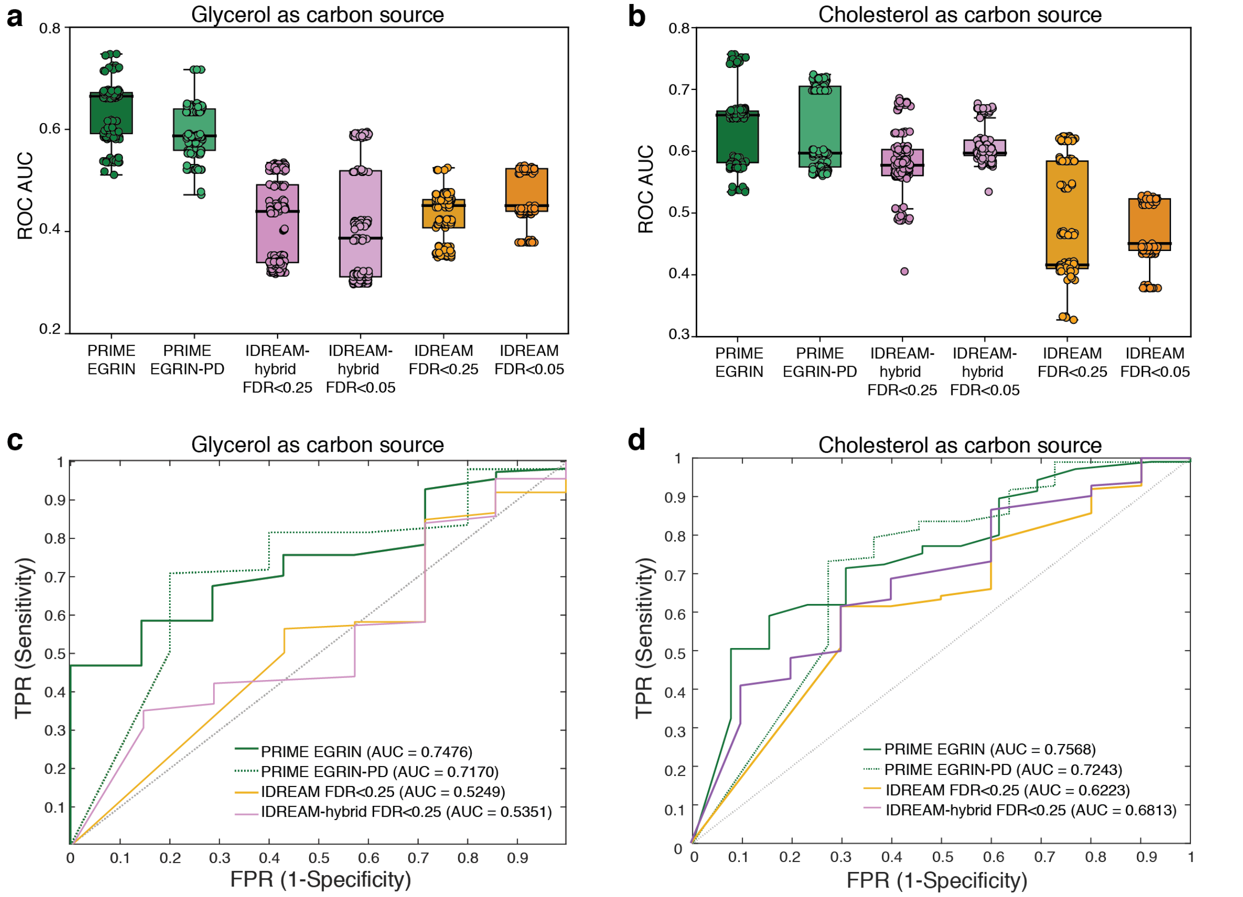

Supplementary Figure 7. Performance assessment of IDREAM-hybrid and its comparison with PRIME and IDREAM. Sensitivity and specificity of PRIME, IDREAM-hybrid, and IDREAM predicted TF essentiality in **a.** glycerol and **b.** cholesterol as determined by LOOCV analysis using the area under the receiver operating characteristic curve (ROC AUC). The statistical significance of these predictions was tested using one way ANOVA and have p value <0.00001. The boxes in the boxplot indicate the upper and lower quartiles of the data and the middle line is the median with the whiskers extending to 1.5× interquartile range. **c** and **d** are representative ROC curves for the performance of PRIME EGRIN and PRIME EGRIN-PD, IDREAM and IDREAM-hybrid in glycerol and cholesterol respectively.

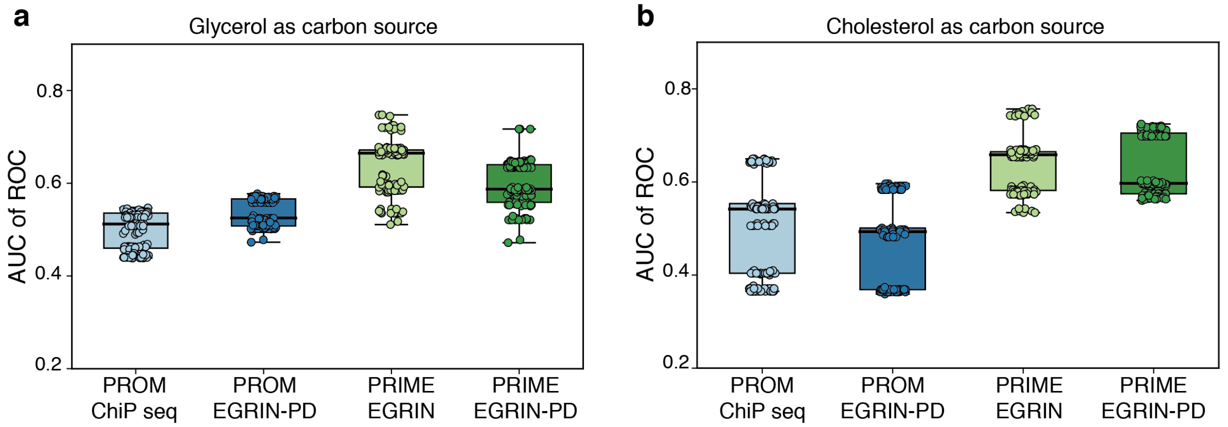

Supplementary Figure 8. Performance assessment of PRIME and PROM with EGRIN-PD network in **a.** Glycerol and **b.** Cholesterol. PRIME outperformed PROM in all comparisons (p value <0.0002 based on one way ANOVA). The boxes in the boxplot indicate the upper and lower quartiles of the data and the middle line is the median with the whiskers extending to 1.5× interquartile range.

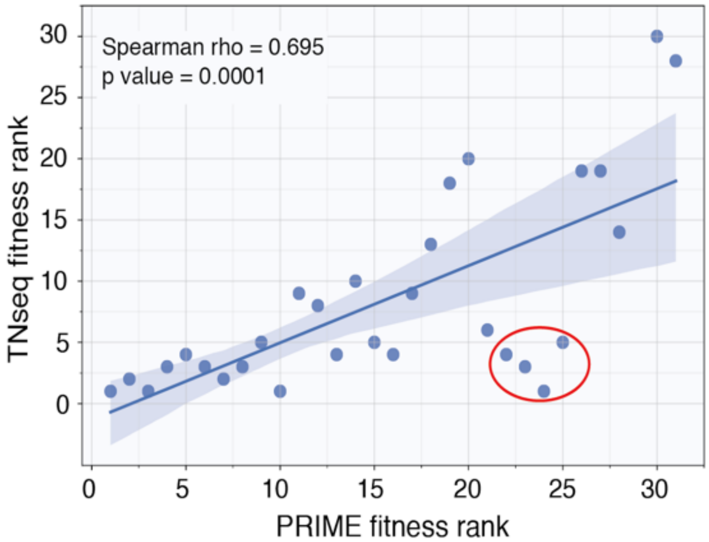

Supplementary Figure 9: TnSeq and PRIME rank correlation. TF fitness from TnSeq experiments were compared with PRIME TF knockout fitness. The red circle highlights TFs that have <10% of target genes as part of metabolic model. These TFs were removed in **Figure 4c**.

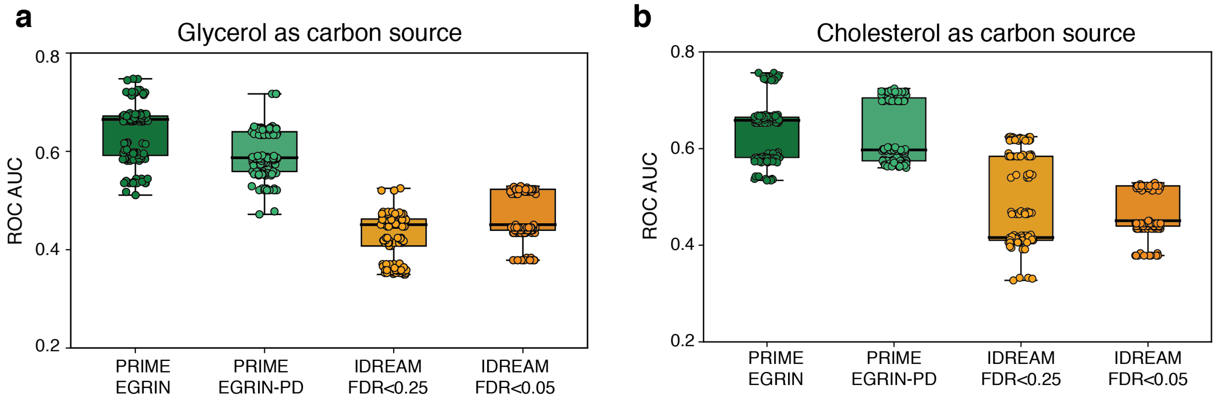

Supplementary Figure 10. Comparison of IDREAM performance at two different thresholds (FDR <0.25 and FDR <0.05) for two conditions (glycerol and cholesterol) and PRIME. The area under the curve (AUC) of the receiver operating characteristic (ROC) curves are plotted as a box plot for all the LOOCV iterations for **a.** Glycerol and **b.** Cholesterol. The statistical significance was calculated using one way ANOVA and the p value was <0.000001. The boxes in the boxplot indicate the upper and lower quartiles of the data and the middle line is the median with the whiskers extending to 1.5× interquartile range. Dots are the data points (n = 142).

Supplementary Table 1:

Summary of all variables used in the PRIME equations:

Variable	Description	Network (From where the variables are derived)
j	TF	EGRIN-Inferelator
i	Gene	Metabolic network
I	Set of all genes coding for the enzymes catalyzing the reaction	Metabolic network
w	Reaction	Metabolic network
$\gamma_{i,w}$	Reaction Influence Factor (RIF)	PRIME
$\beta_{i,j}$	Weights of the TF-gene interaction	EGRIN-Inferelator
X	TF expression	Transcriptome of a given condition
c	Given condition	Present study
B	All conditions	All conditions in the transcriptome compendium
g	Final flux influence factor	PRIME
a	min Flux	Metabolic network
b	max Flux	Metabolic network
v	Flux vector	Metabolic network
